# What about the heart — pediatric ALL survivors show cardiopulmonary limitations in the MinimALL Study

**DOI:** 10.1007/s00431-025-06243-0

**Published:** 2025-06-09

**Authors:** Felix Huber, Isabelle Schoeffl, Nicolas Mueller, Alexander Dierl, Eva-Maria Wild, Nora Naumann-Bartsch, Axel Karow, Ferdinand Knieling, Joachim Woelfle, Sven Dittrich, Felix Anderheiden

**Affiliations:** 1 Department of Pediatric Cardiology, Friedrich-Alexander-Universität Erlangen-Nürnberg, Universitätsklinikum Erlangen, Loschgestrasse 15, 91054 Erlangen, Germany; 2 School of Clinical and Applied Sciences, Leeds Beckett University, Leeds, LS13HE UK; 3 Department of Pediatrics and Adolescent Medicine, Friedrich-Alexander-Universität Erlangen-Nürnberg, Loschgestrasse 15, 91054 Erlangen, Germany

**Keywords:** Pediatric cancer survivors, Cardiopulmonary exercise testing, Cardiotoxicity, Leukemia, Strain imaging

## Abstract

Childhood survivors of acute lymphoblastic leukemia (ALL) and Hodgkin disease (HD) are at risk of long-term cardiopulmonary impairments due to cardiotoxic chemotherapy and inactivity. This study aims to assess the cardiopulmonary fitness and cardiac function of pediatric cancer survivors (PCS) using cardiopulmonary exercise testing (CPET) and echocardiography, including strain imaging, to determine the extent of functional limitations and their underlying causes. This prospective, single-center study included 27 PCS (21 with ALL, 6 with HD) and 27 age-matched healthy controls. Participants underwent echocardiography with strain analysis and CPET on a treadmill to evaluate cardiac function and exercise capacity. Key parameters such as ejection fraction (EF), global longitudinal strain (GLS), E/E′ ratio, peak oxygen uptake ($\dot{V}{O}_{2}peak$), and breathing efficiency were analyzed. PCS exhibited significantly lower $\dot{V}{O}_{2}peak$ compared to controls (38.3 ± 7.7 ml/kg/min vs. 46.4 ± 5.4 ml/kg/min, *p* = 0.001), along with a reduced peak heart rate (*p* = 0.001). Echocardiographic analysis showed that while EF and GLS remained within normal ranges, E/E′ septal was significantly elevated (*p* = 0.001). Pulmonary parameters did not indicate significant ventilatory limitations.

*Conclusion*: Our study underscores the importance of diastolic assessments and advanced echocardiographic techniques in monitoring PCS, with E/E′ septal emerging as a key marker. CPET provides valuable insights into the functional impact of early cardiac changes. Despite signs of diastolic dysfunction at rest, stroke volume and pulmonary function were preserved during exercise, suggesting that detraining may partly contribute to reduced $\dot{V}{O}_{2}peak$.

*Trail registration*: NCT06093334.

**Table:** 

What is Known: • Childhood survivors of ALL and HD are at risk of long-term cardiopulmonary impairments due to chemotherapy-related cardiotoxicity and reduced physical activity. • CPET and echocardiography, including strain imaging, are valuable tools for assessing cardiac function and exercise capacity in this population.
What is New: • Early diastolic dysfunction detected: despite normal EF and GLS, PCS exhibit an elevated E/E′ septal ratio, reinforcing its role as a key marker for early cardiac changes.• Reduced exercise capacity linked to detraining: PCS show significantly lower $\dot{V}{O}_{2}peak$ and peak heart rate, while stroke volume and pulmonary function remain preserved, suggesting detraining as a contributing factor.

## Introduction

Acute lymphoblastic leukemia (ALL) is a major global health challenge, accounting for about 28% of pediatric cancer cases and resulting in millions of deaths [1]. Although survival rates have risen to nearly 90% as a result of treatment advancement [2], the associated cytotoxic chemotherapy can lead to muscle atrophy and cardiac toxicity [3]. Survivors, even 25 years post-treatment, often report declines in mental health, functional impairments, and daily activity limitations [4]. This highlights the growing interest in survivorship, physical fitness, and quality of life post-cancer treatment [5].

Cardiopulmonary exercise testing (CPET) is a key method for assessing cardiopulmonary fitness, offering a comprehensive evaluation of heart, lung, and muscle function [6]. Peak oxygen uptake ($\dot{V}{O}_{2}peak$) is a critical predictor of morbidity and mortality in both healthy individuals and patients [7]. The current body of research remains relatively limited, as most of the investigating studies on pediatric cancer survivors (PCS) have used functional mobility tests and 6-min walk tests [8–10]. In the few studies measuring $\dot{V}{O}_{2}peak$, PCS showed diminished exercise performance compared to age-matched healthy controls [11–13]. However, these studies often did not evaluate the impact of echocardiographic parameters and current fitness levels on $\dot{V}{O}_{2}peak.$

Anthracycline-induced cardiac toxicity can cause myocardial damage, potentially leading to heart failure [14]. While left ventricular ejection fraction (EF) is commonly used for assessing cardiotoxicity, it often detects abnormalities too late for effective intervention [15]. Advanced imaging techniques, such as speckle-tracking for global longitudinal strain (GLS), are increasingly important for identifying subclinical cardiac injuries, indicating that GLS should complement rather than replace EF assessment [16].

In this study, we aim to objectify the cardiopulmonary impairments using CPET and echocardiography — including strain analysis — following chemotherapy in patients with ALL and Hodgkin disease (HD) and to further investigate the underlying causes of these limitations.

## Patients, materials, and methods

This prospective, single-center study was approved by the Ethics Committee of the University of Erlangen-Nuremberg, Germany (23-47_2-B). Written informed consent was obtained from all participants and their legal guardians in accordance with the Declaration of Helsinki. The study is part of a larger research project registered under clinicaltrials.gov as part of the MinimALL Study (NCT06093334):

https://classic.clinicaltrials.gov/ct2/show/NCT06093334?term=MinimALL&draw=2&rank=1.

### Study population

The study included children aged 5 to 17 years diagnosed with ALL or HD who had completed induction therapy or radiotherapy. We selected patients with these two diseases due to their high survival rates and standardized, yet distinct, treatment protocols, including similar anthracycline doses. This allows for a comprehensive comparison of long-term, treatment-related outcomes in PCS. An age- and sex-matched control group of healthy children were selected from our database, with neither a diagnosis of oncological disease nor a history of heart condition (Ethics-Number: 20–528-B). All participants were approached during their routine therapy or follow-up visits.

Exclusion criteria for both groups included pregnancy or lactation, pleural or pericardial effusion, critical conditions requiring respiratory support, symptomatic heart failure, significant thoracic deformities or lung surgery, and injuries preventing participation in exercise tests.

Height and weight were measured using a stadiometer and an electronic scale (Seca 704 S, Hamburg, Germany). We administered a standardized questionnaire to assess sports behavior across different life stages [17].

### Echocardiography

We performed echocardiography using the Vivid E95 cardiovascular ultrasound system (GE Healthcare, Buckinghamshire, UK). Standard echocardiographic views, including parasternal long-axis (PLAX), short-axis (PSAX), apical four-chamber, and two-chamber views, were obtained following established guidelines.

The following standard parameters were measured: EF, fractional shortening (FS), mitral annular plane systolic excursion (MAPSE) and tricuspid annular plane systolic excursion (TAPSE), transmitral early to late diastolic filling velocity ratio (E/A), the ratio of early transmitral flow velocity to early diastolic mitral annular velocity (E/E′) of the lateral and septal mitral annulus and maximal velocity (Vmax) of the left ventricular outflow tract (LVOT). Tissue Doppler was used for measuring E′.

Furthermore, the size of the left ventricle (LV) was recorded by measuring the mean internal dimensions of the ventricle during end-diastole (LVIDd) and end-systole (LVIDs) in parasternal short and long-axis views.

Strain analysis was performed to assess myocardial deformation. GLS of the left ventricle, left atrium, and right ventricle was quantified using dedicated software (Syngo Dynamics, Siemens Healthineers©, Munich, Germany).

Additionally, Relative Wall Thickness (RWT) and the Sphericity Index (SI) [18] were measured:

$$\begin{array}{cc}RWT= \frac{2\;x\;LV\;Posterior\;Wall\;Thickness}{LVIDd}& SI=\frac{LVIDd\;in\;PSAX}{LV\;length\;(enddiastolic\;four-chamber)}\end{array}$$

The same experienced sonographers performed all echocardiographic examinations. All echocardiographic values were compared with normal (control) values using a z-score calculator: https://www.pedz.de/de/herz.html [19].

### Cardiopulmonary exercise test

Verbal encouragement was provided to all participants, and tests were performed until exhaustion. All tests were executed by the same two researchers.

During CPET, electrocardiography (ECG), heart rate (HR), and oxygen pulse, reflecting oxygen consumption per heartbeat, were monitored using a twelve-lead ECG (Custo®, Ottobrunn, Germany) and a Nonin PureSAT Oximeter (Nonin® Medical, Inc., Plymouth, MN, USA). To achieve maximal exertion, we used different stepwise treadmill protocols adapted to the participant’s age and physical fitness (COSMED T170, COSMED, Italy). Mostly, the initial individual speed was set for 2 min, followed by two km/h increments every 2 min until a Respiratory Exchange Ratio (RER) of 1.0 was reached. After this point, speed was increased by one km/h per stage up to volitional fatigue. For younger children, a modified protocol with smaller increments of velocity (v) (0.5 km/h) and gradual elevation increases were used to accommodate their physical capacity and enhance motivation. Gas exchange was measured continuously using a compact respiratory valve with minimal dead space (88 ml), a mouthpiece, and headgear individually adjusted for each participant (Metalyzer 3B, Cortex, Leipzig, Germany). Data were collected on a breath-by-breath basis, and results were averaged every 15 s.

To objectify $\dot{V}{O}_{2}peak$, we applied the following criteria, requiring at least two of the following conditions: (1) peak HR within 5% of the age-predicted maximum, (2) RER ≥ 1.0, and (3) volitional exhaustion [20, 21]. Given the difficulty of achieving high RER values in children during treadmill-based CPET, an RER threshold of 1.00 was deemed sufficient. Normal values from Kalden et al. [22] and Bongers et al. [23] were used for children aged below 8 and 9–16 years, respectively, with adult norms [24] applied to those aged 16 and older.

The V-slope method, as described by Beaver et al. [25], was used to determine ventilatory thresholds (VT1 and VT2). The oxygen uptake efficiency slope (OUES) was also calculated using linear regression analysis between $\dot{V}{O}_{2}$ and minute ventilation ($\dot{V}E$) during incremental exercise [26, 27], starting 1 min after the onset of exercise and ending at VT2. Additionally, the $\dot{V}{O}_{2}/P-slope$ was determined as the linear relationship between $\dot{V}{O}_{2}$ and external work rate (in Watt). The ventilatory response during exercise was assessed by plotting $\dot{V}E$ against carbon dioxide elimination ($\dot{\text{V}}C{O}_{2}$), with the slope $\dot{V}E/\dot{V}C{O}_{2}$ derived from the regression line, excluding data beyond the ventilatory compensation point. To objectify if study and control patients are equally able to recover, the half-time recovery of $\dot{V}{O}_{2}$ (${T}_{1/2}\dot{V}{O}_{2}$) was recorded during the off-transient phase after $\dot{V}{O}_{2}peak$. Heart rate recovery (HRR) was monitored during the first minute of the recovery phase.

Standard ECG recordings during rest were analyzed for rhythm abnormalities, and LV hypertrophy was assessed using the Sokolow-Lyon index.

### Statistical analysis

Data were collected in Microsoft Excel (Version 2000), and statistical analyses were performed using SPSS (Version 29.0.1.0, SPSS Inc., Chicago, IL). Results are presented as means ± standard deviations. Normal distribution was assessed using the Kolmogorov–Smirnov test, and homogeneity of variance was evaluated using Levene’s *F*-test. For normally distributed data, group differences were analyzed using unpaired *t*-tests, while non-parametric data were assessed with Wilcoxon or Mann–Whitney *U*-tests. Non-parametric analysis produced similar results to the parametric analysis, therefore, only the results from the student *t*-test are presented. Statistical significance was defined as *p* < 0.05. The Pearson correlation coefficient was employed to analyze the bivariate correlation between independent variables and *V̇*O₂*peak*. For the comparison of ordinal and nominal variables, the Fisher’s exact test was utilized. We also performed non-parametric tests since the study group was too small for objectifying the results.

## Results

### Anthropometric and clinical data

**Fig. 1:**
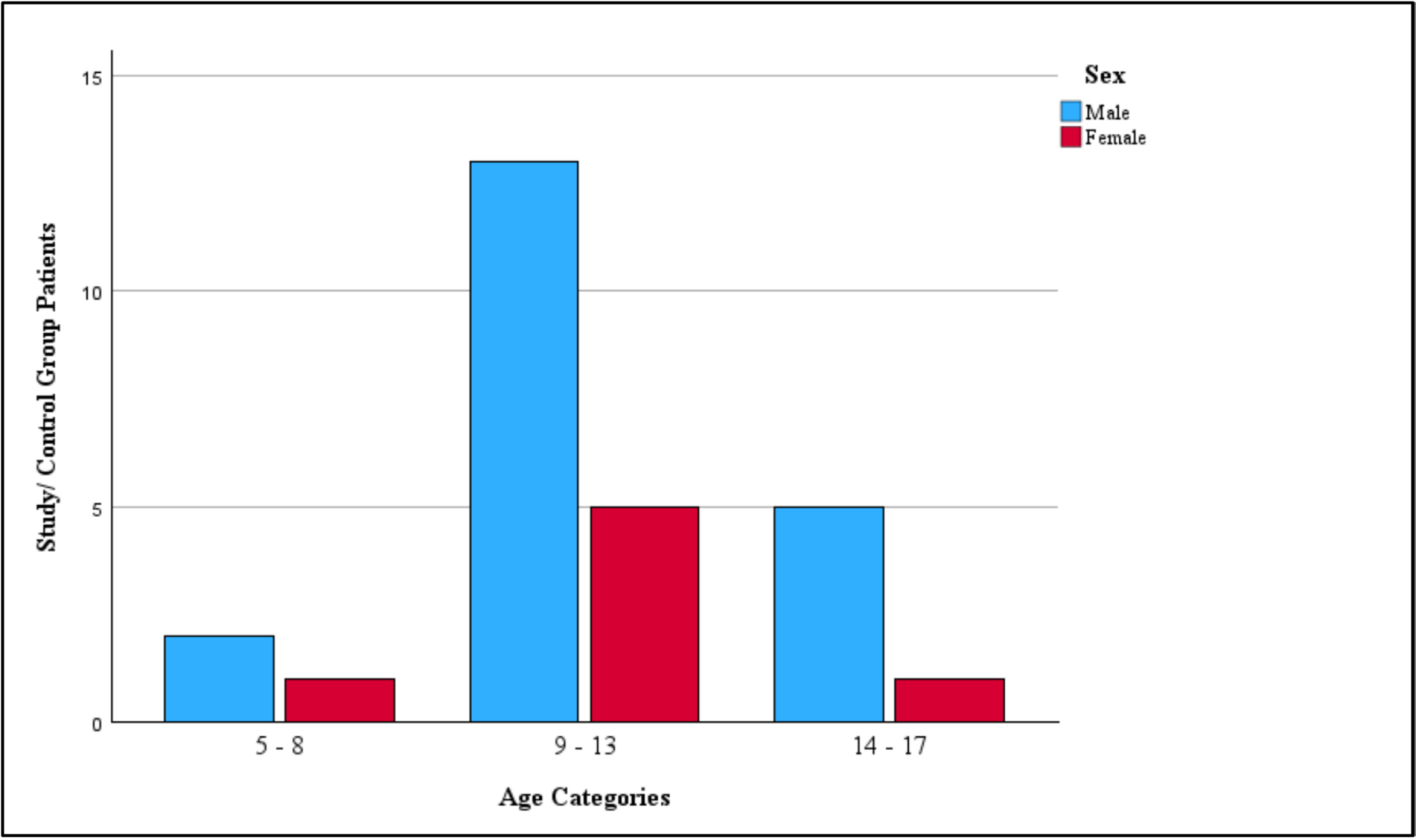
Sex-specific distribution of patients across age categories in the study population

The study included 27 participants in the study and 27 in the control group. Among the cancer survivors, 21 were diagnosed with ALL, while six were survivors of HD. There were no significant differences between study and control group patients with respect to age, height, weight, or sex (Table 1). A more detailed age distribution is demonstrated in Fig. 1. The control group, identical to the study group in age and sex distribution, only performed CPET and did not receive echocardiographic evaluation.

**Table 1:** Baseline characteristics and physical activity profile in means and standard deviation

	Study group	Control group	*p*-value
*n*	27	27	
Age (y)	11.7 ± 2.9	11.7 ± 3.0	0.961
Weight (kg)/*Z*-score	43.2 ± 15.6/+ 0.10	40.4 ± 13.2/− 0.10	0.469
Height (cm)/*Z*-score	150.7 ± 17.0/− 0.06	152.2 ± 17.0/+ 0.06	0.675
Body mass index (kg/m^2)^/*Z*-score	18.7 ± 3.9/+ 0.24	17.1 ± 2.8/− 0.24	0.077
Body surface area (m^2^)	1.3 ± 0.3	1.3 ± 0.3	0.793
Physical activity as a toddler (h/week)	2.9 ± 2.2	2.6 ± 2.1	0.619
Physical activity as a schoolchild (h/week)	4.5 ± 2.8	4.8 ± 3.2	0.740
Physical activity at current age (h/week)	4.0 ± 3.0	5.4 ± 3.9	0.203
Self-perception of physical fitness to peers is better/same/worse	9.0%/44.5%/44.5%	31.8%/63.6%/4.5%

In our study group, PCS were tested in mean 4.23 ± 3.32 years after the beginning of the respective chemotherapy, and did receive cumulative anthracycline dosages (mean 180.3 ± 48.3 mg/m^2^). In accordance with current guidelines [28], we calculated the dosages of doxorubicin and their equivalents for each patient and in summary categorized the cohort in three risk groups (Table 2). In addition, ten study patients have been treated with cranial, pulmonary (ALL), or mediastinal (HD) radiation.

**Table 2:** Distribution of study population by cumulative anthracycline dosage and radiotherapy

Risk group	Number of patients	Cumulative anthracycline dose (mg/m^2^)	Radiotherapy
Low risk	2	≤ 100	-
Moderate risk	15	100–250	7
High risk	3	≥ 250	3
Total	27	180.3 ± 48.3	10

In our questionnaire, both cohorts reported very similar levels of physical activity as a toddler and school child. At present, PCS spend an average of 1.4 h less per week on sports in contrast to their peers, but this difference did not reach significance. A notably higher proportion of the study group (64%) commuted to school by bus or car instead of bike or by foot, compared to about 43% in the control group. Being questioned about their self-perception to fitness, almost half (44,5%) of the survivors believed their exercise capacity was worse than their peers.

### Echocardiography

The measurements from the echocardiographic studies are represented in Table 3 along with the respective normal values for all recorded parameters.

**Table 3:** Results from echocardiography and electrocardiogram in mean ± standard deviation

	Study group	Norm (mid) values [19]	*p*-value
EF (%)	62.8 ± 7.3	> 55	
FS (%)	34.9 ± 6.0	> 28	
MAPSE (mm)	13.6 ± 2.4	14.2 ± 0.9	0.254
TAPSE (mm)	21.5 ± 3.3	21.3 ± 1.7	0.833
E/A	2.0 ± 0.4	1.9 ± 0.0	0.300
E/E′ septal	6.8 ± 1.5	5.1 ± 0.5	0.001
E/E′ lateral	5.6 ± 1.1	5.2 ± 0.5	0.055
Vmax LVOT (m/s)	1.2 ± 0.1	< 1.8	
LVIDd (mm)/*Z*-score	42.2 ± 4.0/−0.22	42.8 ± 4.2/+ 0.06	0.372
LVIDs (mm)/*Z*-score	27.9 ± 4.3/+ 0.19	27.7 ± 3.9/+ 0.17	0.837
LV-strain	−18.8 ± 2.3	< − 16	
LA-strain	42.2 ± 13.0	> 35	
RV-strain	−23.0 ± 3.7	< − 20	
Sphericity index	0.64 ± 0.10	0.6–0.7	
RWT	0.34 ± 0.11	0.32–0.42	
Sokolow-Lyon-index (mV)	2.50 ± 0.67	< 3.5	

In terms of LV function, almost all study patients had an EF greater than 55% and an FS greater than 28%. Both MAPSE and TAPSE were within normal ranges. Septal E/E′ was significantly higher in PCS than norm values (*p* = 0.001) while E/E′ lateral (*p* = 0.055) did almost approach statistical significance. Septal E/E′ showed abnormal age-adapted — thus pathological — results in more than half of the subjects (51.9%). The third diastolic component, E/A, showed normal and comparable values in both groups.

We also recorded GLS values of the LV for all subjects. All but two patients had normal GLS of the LV. Left atrial (LA) and right ventricular (RV) strain were within the normal range. Also, LV size was similar between groups. The calculations of the SI and RWT did not differ significantly between normal and recorded values; however, in ten PCS, SI values were slightly lower than normal.

### Cardiopulmonary exercise test

The results of the CPET are summarized in Table 4. Both groups achieved a similar RER and exercise time. All participants achieved RER values higher than 1.0, suggesting a good effort even though the age of the study group is young, and testing in this age group can sometimes be challenging. We used strong verbal encouragement to achieve these results. There were no signs of arrhythmia or ischemia during all procedures. Interestingly, the study group demonstrated a significant lower peak HR compared to the control group (*p* = 0.001), alongside a significant lower Vmax at $\dot{V}{O}_{2}peak$ values (*p* = 0.005).

**Table 4:** Results from the cardiopulmonary exercise test in means ± standard deviation

	Study group	Control group	*p*-value
Peak RER	1.04 ± 0.07	1.04 ± 0.05	0.982
Exercise time (seconds)	887.1 ± 214.3	977.9 ± 153.2	0.079
Predicted $\dot{V}{O}_{2}peak$ (%)	80.3 ± 16.5	86.5 ± 15.8	0.168
$\dot{V}{O}_{2}VT1$(ml/kg/min)	25.1 ± 6.9	28.7 ± 6.8	0.060
Vmax $\dot{V}{O}_{2}peak$ (km/h)	9.5 ± 2.3	11.2 ± 1.9	0.005
$\dot{V}Emax$(l/min)	62.6 ± 28.4	70.7 ± 21.9	0.250
Peak BF (breaths/min)	53.4 ± 9.4	58.5 ± 10.5	0.067
Peak oxygen pulse (ml)	9.3 ± 4.3	9.6 ± 2.9	0.740
Peak HR (beats/min)	184.3 ± 12.4	196.5 ± 9.8	0.001
HRR (beats/min)	−36.5 ± 8.4	−36.0 ± 15.1	0.093
$\dot{V}{E/\dot{V}CO}_{2}$-Slope	34.0 ± 4.1	35.1 ± 4.7	0.395
$\dot{V}{O}_{2}/P$-Slope (ml/min/W)	12.6 ± 3.9	14.7 ± 3.2	0.043
OUES (L/min)	1.9 ± 0.8	2.1 ± 0.6	0.476
OUES (ml/min/kg)	44.3 ± 9.5	52.4 ± 7.6	0.001
${T}_{1/2}\dot{V}{O}_{2}$(sec.)	114.0 ± 50.9	114.5 ± 42.8	0.971
HR at rest (beats/min)	96.2 ± 12.1	101.4 ± 11.3	0.111
petCO2 VT1	35.6 ± 3.3	35.4 ± 3.0	0.832
$\dot{V}{E/\dot{V}CO}_{2}VT1$	27.2 ± 2.2	28.1 ± 1.6	0.095
$\frac{\dot{V}{E/\dot{V}CO}_{2}VT1}{\text{petCO}2\text{ VT}1}$	0.77 ± 0.12	0.80 ± 0.11	0.373

**Fig. 2:**
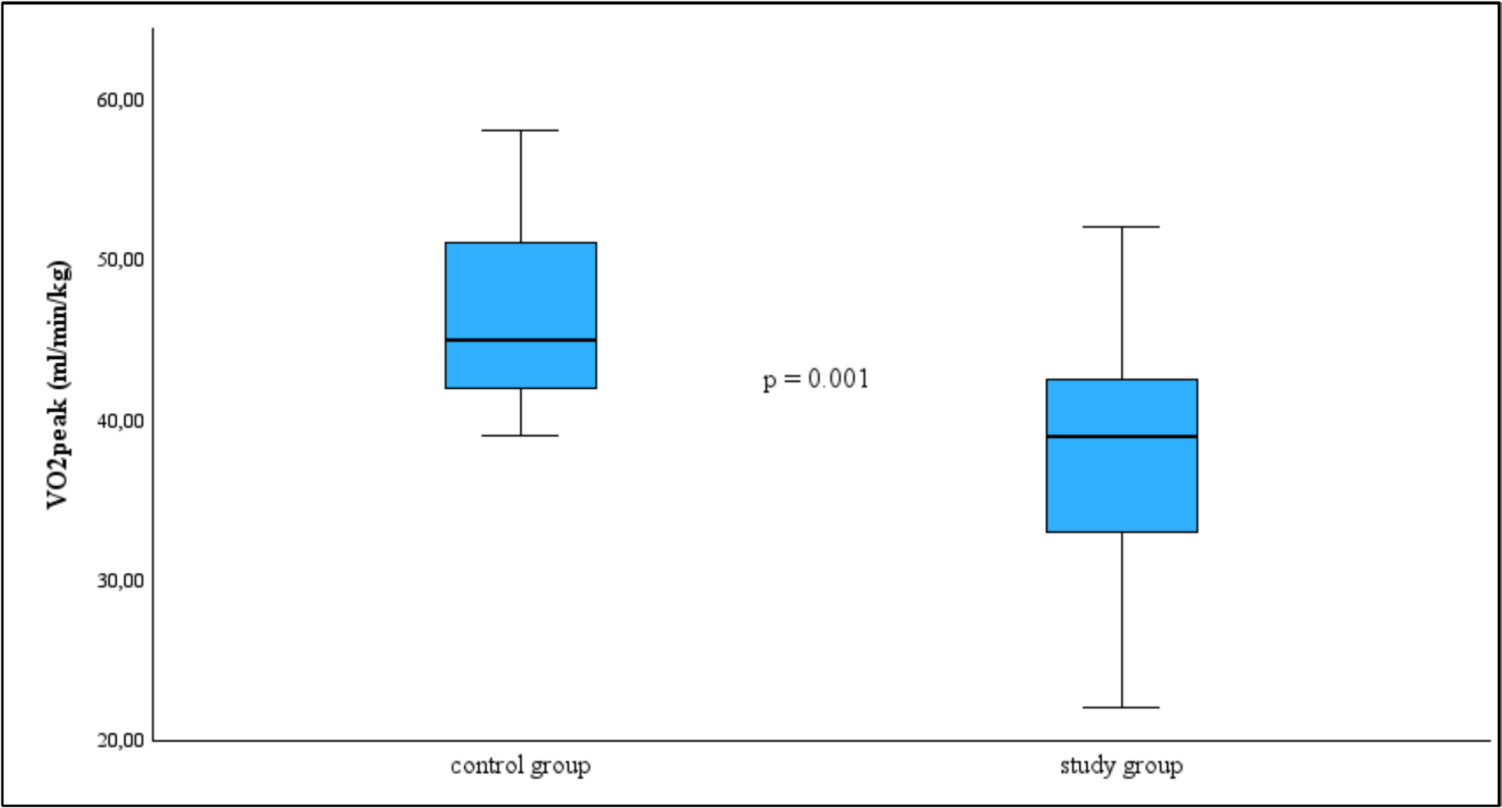
Median and interquartile range of $\dot{V}{O}_{2}peak$ for control and study group

As shown in Fig. 2, there was also a highly significant difference in $\dot{V}{O}_{2}peak$ with the study group achieving lower values compared to the controls (38.3 ± 7.7 ml/kg/min vs. 46.4 ± 5.4 ml/kg/min, *p* = 0.001). In more detail, 67% of the study group and 37% of the control group exhibited $\dot{V}{O}_{2}peak$ < 85% of their predicted $\dot{V}{O}_{2}peak$, although percent predicted $\dot{V}{O}_{2}peak$ was not statistically different between groups. Both female and male participants with PCS showed significantly lower $\dot{V}{O}_{2}peak$ values compared to sex-matched controls, with a more pronounced reduction observed in females (32.0 ± 6.35 vs. 44.0 ± 4.24 ml/kg/min, *p* = 0.001) than in males (40.55 ± 6.96 vs. 47.37 ± 5.61 ml/kg/min, *p* = 0.002).

Other cardiac parameters, such as peak oxygen pulse, HRR, and HR at rest, were comparable between the study and control groups. There was no significant difference in maximal $\dot{V}E$, $\dot{V}E$ at VT1 and the slope $\dot{V}E/\dot{V}C{O}_{2}$ between the groups. When adjusted for body mass (ml/min/kg), the OUES was significantly lower in the study group compared to controls. VT1 for end-tidal carbon dioxide pressure (petCO2) or $\dot{V}E/\dot{V}C{O}_{2}$, nor its ratio, was found to be different between the groups. No correlations were found between cardiac and spiroergometric parameters, only the number of days since chemotherapy and $\dot{V}{O}_{2}peak$ showed a tendency towards positive correlation (*p* = 0.057).

## Discussion

Summarizing our findings, echocardiographic parameters were largely within normal ranges. Still, over half of PCS had elevated septal E/E′, suggesting subclinical diastolic dysfunction. CPET revealed significantly reduced $\dot{V}{O}_{2}peak$ and lower peak HR in the study group, indicating diminished exercise capacity.

### Physical activity

Our well-matched study groups showed no significant difference in past sports activity, but fewer current sports hours in PCS suggest a lasting impairment even years after treatment. Most PCS in similar studies did not meet physical activity guidelines, leading to sedentary behavior and significantly lower fitness [29–31]. The higher use of passive transport (car/bus) in the study group may indicate persistent parental caution or less inclination by PCS to participate in activity after intensive treatment. In our survey, 44.5% of PCS perceived their fitness as lower than their peers, which not only impacts their ability to participate in activity but may also affect self-esteem. Research also links ALL treatment and associated periods of isolation to increased mental health risks [32, 33], including depressive symptoms and fatigue [34]. Interventions targeted at improving activity levels and physical fitness may in turn benefit psychological health and motivation.

### Echocardiography

Echocardiography is the standard way to assess cardiac function post-ALL [35, 36]. While systolic function measures were within normal ranges, a significant proportion of patients demonstrated early signs of diastolic dysfunction through elevated E/E′ septal values. This finding aligns with existing literature suggesting that diastolic dysfunction often precedes systolic impairment [37–39], with E/E′ septal exhibiting greater vulnerability in certain cases [40]. Although no significant abnormalities were observed in other diastolic parameters in our study, previous research documented impairments in E/E′ lateral [40] and E/A ratio [39, 41]. Interestingly, Ryerson et al. [41] conducted echocardiography on anthracycline-treated PCS at rest and directly after exercise. At-rest abnormalities (elevated E/E′ septal and decreased E/A parameters) were detected in high-risk patients and not in low-risk PCS, but after exercise abnormalities were not noticed in either group. Strain imaging has been increasingly recognized for its ability to detect subclinical cardiac abnormalities that may not be captured by conventional echocardiographic methods [16, 38, 42, 43]. In fact, GLS can reveal early myocardial changes, especially in patients exposed to higher doses of anthracyclines or radiotherapy [44]. In our study, strain imaging of both ventricles and the left atrium showed no significant abnormalities. Given that anthracycline-induced cardiotoxicity is typically dose-dependent, with a cumulative threshold of > 240 mg/m^2^ associated with significant reductions in left ventricular GLS [44, 45], the absence of relevant strain abnormalities in our study may reflect the relatively lower risk in our study population (mostly < 240 mg/m^2^). Another reason for the normal GLS findings could be attributed to the relatively short-term period after the start of chemotherapy in our assessed cohort (mean 4.23 ± 3.32 years after the beginning of chemotherapy). Similarly, other studies have shown diastolic abnormalities in the earlier phase post-chemotherapy [37, 41], whereas systolic impairments are largely reported in later follow-ups [43, 46, 47]. Furthermore, patients who received mediastinal radiotherapy (HD) might remain at higher risk for GLS reductions, as demonstrated by Yu et al. [46], suggesting that GLS imaging should be employed as a critical tool in the long-term surveillance of high-risk patients. We tested ten patients that underwent pulmonary, cranial, or mediastinal radiation, but no correlation to relatively worsened GLS could be observed. The relationship between LV size, mass, and overall cardiac function is also noteworthy. Recent findings by Masood et al. [48] emphasize that reduced LV dimensions can limit stroke volume and consequently $\dot{V}{O}_{2}peak$, potentially impairing exercise capacity. In our study we showed that LV dimensions were similar and overall cardiac function (EF and FS) was preserved. In addition, we did not observe significant signs of hypertrophic remodeling in our cohort compared to age-adapted norm values. Despite the absence of hypertrophic changes, some abnormalities in the left ventricular SI were observed in the study group. Abnormal SI values have been associated with early cardiac remodeling, as demonstrated in studies on adult patients with coronary artery disease [49] and acute myocarditis [50]. Although pediatric-specific data are limited, it is plausible that these structural changes represent an early adaptive response aimed at preserving cardiac output in the face of diastolic dysfunction.

Our echocardiographic findings contribute to the growing body of evidence that PCS, particularly those treated with anthracyclines, show signs of increased risk for early cardiac remodeling and diastolic dysfunction [40, 51]. However, the long-term impact of these echocardiographic abnormalities on exercise capacity is unclear, as indicated by Cifra et al. [52], who found that even after 10 years of follow-up, survivors with slight diastolic and right ventricular strain abnormalities demonstrated no significant impairments in dynamic exercise response.

To further investigate the relationship between cardiac function and exercise capacity, we conducted CPET on all study and control participants.

### Cardiopulmonary exercise testing

Both groups reached comparable levels of peak exertion, reflected by similar RER and HRR. However, the PCS group exhibited significantly lower peak HR, consistent with findings from other studies [13, 53], which have identified chronotropic incompetence (CI) as a key factor limiting exercise performance in cancer survivors. The exact cause of CI, an independent predictor of cardiovascular events [54], is unclear but may involve beta-adrenergic receptor insensitivity [55], chemotherapy-induced autonomic dysregulation, and general deconditioning. PCS in our study demonstrated significantly lower $\dot{V}{O}_{2}peak$, a result that aligns with numerous similar studies [11, 13, 48, 56]. For instance, Foulkes et al. [13] performed CPET on 20 PCS (ages 8–24), on average 4.4 years after their cancer diagnosis, following treatment with anthracyclines and, in some cases, radiation therapy. In their cohort, 60% of the survivors had a $\dot{V}{O}_{2}peak$ below 85% of the predicted value, according to the American Journal of Respiratory and Critical Care Medicine’s definition [57]. Similarly, we assessed PCS 4.2 years after beginning chemotherapy and discovered 67% exhibiting reduced exercise capacity ($\dot{V}{O}_{2}peak$ < 85% of predicted $\dot{V}{O}_{2}peak$). The absence of significant differences in oxygen pulse and heart rate reserve (HRR) suggests that central cardiovascular function and ventilatory efficiency may be relatively preserved in PCS patients. Notably, the diastolic dysfunction identified at rest through echocardiography did not seem to impair stroke volume during exercise, a finding consistent with previous studies [52, 58]. For instance, De Caro et al. [58] demonstrated that PCS with subclinical cardiac changes, such as reduced heart wall dimensions, maintained a normal myocardial response to exercise. This suggests that while resting diastolic abnormalities may indicate early signs of cardiac remodeling, they do not necessarily translate into impaired functional capacity during physical exertion. While our data indicates preserved stroke volume, indirectly estimated via oxygen pulse, this contrasts with findings by De Souza et al. [59], who used stress echocardiography and reported a blunted stroke volume index response to exercise in PCS. The discrepancy may be explained by differences in methodology and treatment intensity, as our patients received lower anthracycline doses. Still, although peak oxygen pulse was comparable between our groups, the lower peak HR in PCS indicates that total cardiac output was likely reduced. According to Fick’s equation ($\dot{V}{O}_{2}$ = cardiac output × arteriovenous oxygen difference), this reduction in HR could partially explain the diminished $\dot{V}{O}_{2}peak$ in PCS. A further relevant parameter is the $\dot{V}{O}_{2}/P$ slope, reflecting the efficiency of oxygen uptake in relation to external work rate. Although significant group differences were modest and values only slightly below the pathological threshold of 10 ml/min/W [60], our findings may point to early, subclinical limitations in oxygen transport or utilization. The lack of significant differences in pulmonary variables, including V̇E peak, V̇E at VT1, breathing frequency (BF), and absolute OUES values, suggests that pulmonary function was not the primary limiting factor for reduced exercise tolerance in the study group. However, when the OUES was normalized to body mass (ml/min/kg), a significant reduction was observed in the study group, indicating lower ventilatory efficiency relative to body size. While this supports that maximal effort was reached during testing, it also points to subtle cardiopulmonary inefficiencies that may not be captured by conventional pulmonary parameters alone. Considering these findings, we further examined potential patterns of dysfunctional breathing. Numerous studies have recognized the increased ventilatory equivalent for carbon dioxide ($\dot{V}E/\dot{V}C{O}_{2}$-Slope) as a marker for a diffusion-perfusion deficit of the lung in CPET [61, 62]. This non-invasive parameter for ventilatory efficiency is associated with several cardiopulmonary diseases, for example heart failure [63], pulmonary arterial hypertension [64] or dysfunctional breathing [65]. Given our non-significant results between PCS and healthy children, we could not indicate this ventilatory mismatch of diffusion and perfusion being the cause of lower $\dot{V}{O}_{2}peak$. Parallel to our study, Tsuda et al. could not detect alterations in the $\dot{V}E/\dot{V}C{O}_{2}$-slope in order to substantiate diminished $\dot{V}{O}_{2}peak$ in PCS [56]. Instead, they also suggested limited stroke volume reserve to be the underlying cause of pulmonary deficiency in PCS. As E/E′ septal was elevated in our study group, this could affect the right ventricle and thus affect subclinical pulmonary function. Therefore, we additionally calculated an equivalent ratio for pulmonary arterial hypertension (PAH), based on previous studies [66, 67]. In contrast to these studies, we could not detect indices for PAH in our patients, which is in line with current literature as only rare cases of PAH after leukemic diseases in children are described [68, 69]. The third component of CPET, peripheral muscle limitations, is difficult to measure as specific markers are missing and muscular biopsy is not routinely performed. However, in most similar studies description of peripheral muscle limitation is assumed after excluding ventilatory and cardiac limitations [61, 70]. Given that muscular atrophy is a well-documented side effect of chemotherapy [71], it likely contributes to the reduced exercise capacity observed in PCS. Structured exercise interventions for PCS might be a reasonable approach to improve muscle performance and enhance cardiovascular response, including maximal heart rate [72].

## Limitations

This study has several limitations that should be acknowledged. First, the small sample size limits the generalizability of our findings and may reduce the statistical power to detect subtle effects. Second, the questionnaire used in this study was relatively basic, neither including detailed information on pre- and post-treatment exercise behavior nor focusing on the various chemotherapy regimens nor considering seasonality. Furthermore, while quality of life is a critical factor in assessing long-term outcomes in cancer survivors, our study did not include a psychological analysis.

## Conclusion

Our study highlights the critical role of diastolic assessments, advanced echocardiographic techniques, and CPET testing in the long-term monitoring of PCS. Our study indicates that septal E/E′ and $\dot{V}{O}_{2}peak$ are reduced in PCS. The mechanism by which septal E/E′ affects $\dot{V}{O}_{2}peak$ is unclear. Our findings suggest that both a reduction in cardiac output and/or peripheral deconditioning may contribute to the reduced $\dot{V}{O}_{2}peak$ seen in our group of PCS. Exercise interventions may effectively improve $\dot{V}{O}_{2}peak$ in this population by targeting both cardiac output and peripheral deconditioning in this population.

## Data Availability

No datasets were generated or analysed during the current study.

## Abbreviations

ALL: Acute lymphoblastic leukemia
BF: Breathing frequency
CPET: Cardiopulmonary exercise testing
E/A: Early transmitral flow to late diastolic filling velocity ratio
E/E′: Early transmitral flow to early diastolic mitral annular velocity ratio
ECG: Electrocardiography
EF: Ejection fraction
FS: Fractional shortening
GLS: Global longitudinal strain
HD: Hodgkin disease
HR: Heart rate
HRR: Heart rate recovery
LA: Left atrium
LV: Left ventricle
LVIDd: Left ventricular internal dimension during diastole
LVIDs: Left ventricular internal dimension during systole
LVOT: Left ventricular outflow tract
MAPSE: Mitral annular plane systolic excursion
OUES: Oxygen uptake efficiency slope
PCS: Pediatric cancer survivors
petCO2: End-tidal carbon dioxide pressure
RER: Respiratory exchange ratio
RV: Right ventricle
RWT: Relative wall thickness
SI: Sphericity index
${T}_{1/2}\dot{V}{O}_{2}$: Half time recovery of $\dot{V}{O}_{2}$
TAPSE: Tricuspid annular plane systolic excursion
v: Velocity
$\dot{V}C{O}_{2}$: Carbon dioxide elimination
$$\dot{V}E$$: Minute ventilation
$$\dot{V}E/\dot{V}C{O}_{2}$$: Breathing efficiency
$$\dot{V}{O}_{2}$$: Oxygen uptake
$\dot{V}{O}_{2}peak$: Peak oxygen uptake
$\dot{V}{O}_{2}/P$: Oxygen uptake efficiency relative to increasing workload
Vmax: Maximal velocity
VT1 and VT2: Ventilatory threshold 1 and 2
